# Paliperidone-induced neuroleptic malignant syndrome: A case report

**DOI:** 10.1097/MD.0000000000041171

**Published:** 2025-03-07

**Authors:** Gonca Ayse Unal

**Affiliations:** a Clinic of Psychiatry, Mersin Sehir Egitim ve Arastirma Hastanesi, Toroslar, Mersin, Turkey.

**Keywords:** case report, long-acting injection, neuroleptic malignant syndrome, paliperidone palmitate, schizophrenia, second generation antipsychotics

## Abstract

**Rationale:**

Neuroleptic malignant syndrome (NMS) is a rare, life-threatening complication of neuroleptic (antipsychotic) medications. Paliperidone is an atypical antipsychotic used in the treatment of schizophrenia. While current evidence suggests that atypical oral antipsychotics have a lower incidence of NMS compared to typical oral antipsychotics, there is limited information available on the incidence and management of NMS associated with long-acting injectable (LAI) antipsychotics. More case studies are needed to fully understand the true incidence of NMS associated with LAI atypical antipsychotics. This report aims to present our experience with a patient with schizophrenia who developed NMS as a result of taking LAI paliperidone, an atypical antipsychotic.

**Patient concerns:**

A 63-year-old male diagnosed with schizophrenia was brought to the emergency room with complaints of high fever, muscle contraction, tremors, urinary and fecal incontinence, and a change of consciousness, which started 2 days after the first LAI paliperidone injection. During the psychiatric examination, the patient exhibited tremors, a high fever of 39.5°C, and muscle rigidity. He was confused and had impaired orientation. The patient’s creatine kinase, white blood cell count, aspartate aminotransferase, and alanine aminotransferase levels were measured at 1895 U/L, 13750/mm^3^, 55 IU/L, and 45 IU/L, respectively. The electrocardiogram showed sinus tachycardia.

**Diagnoses:**

The patient’s psychiatric examination, vital signs, and laboratory results were consistent with NMS.

**Interventions:**

The patient received treatment with bromocriptine and amisulpride for 13 days.

**Outcomes:**

With this treatment, the patient’s delirium improved and the patient became stable. The levels of creatine kinase, white blood cell count, aspartate aminotransferase, and alanine aminotransferase decreased to normal values. The patient was discharged from the hospital after 14 days of treatment.

**Lessons:**

Although NMS is not very common in patients using antipsychotics, it is important because it is an emergency condition that can result in death if not treated appropriately. Clinicians should consider NMS in patients taking atypical antipsychotics such as paliperidone.

## 1. Introduction

Neuroleptic malignant syndrome (NMS) is one of the important neurological complications associated with psychiatric drugs. NMS was first described in the 1960s due to the use of neuroleptic drugs.^[1]^ Today, neuroleptics, also called antipsychotics, are used in the treatment of many psychiatric diseases. NMS arises as an idiosyncratic reaction to the antipsychotic agent used, therefore it is an unpredictable iatrogenic neurologic emergency condition.^[1]^ Most cases of NMS are associated with antipsychotics, although it has also been reported in association with other drugs such as lithium, antidepressants, metoclopramide, and also following abrupt discontinuation of dopamine agonists in Parkinson disease.^[2]^ The incidence of NMS has been reported between 0.02% and 3% among patients receiving antipsychotic therapy.^[1]^ NMS associated with first-generation (conventional or typical) antipsychotics is a known side effect and the risk of NMS is higher, especially in those using high potency antipsychotic drugs (especially haloperidol).^[2]^ It has been suggested that second-generation (atypical) antipsychotics carry a lower risk of NMS than typical antipsychotics.^[3]^ However, it is reported that more case information is needed to know the true incidence of NMS associated with atypical antipsychotics.^[4]^ Paliperidone, the major active metabolite of risperidone, has been shown to effectively treat schizophrenia, bipolar disorder, and other related disorders with psychotic symptoms. It is one of the newest second-generation antipsychotics on the market.^[5]^ As publications on atypical antipsychotic-related NMS cases increase, the data to be obtained will contribute to providing the necessary information for a more accurate clinical approach to this group of patients. In this report, a case of NMS associated with paliperidone, an atypical antipsychotic is presented.

## 2. Case report

A 63-year-old male patient who was followed up with a diagnosis of schizophrenia was admitted to the emergency department by his relatives with muscle contraction, tremor, urinary and fecal incontinence, changes in consciousness and high fever symptoms (39.5 °C oral). Pulse rate was 120; blood pressure was 140/90 mm Hg. It was learned that these symptoms had begun after 2 days following paliperidone long-acting injectable antipsychotic injection 100 mg (paliperidone palmitate 156 mg) for the first time. It was learned that the patient’s treatment was changed to paliperidone palmitate once a month, 2 days ago, due to the patient’s irregular use of oral aripiprazole 15 mg daily and quetiapine 100 mg daily for the last 6 months. The patient had no history of drug or alcohol use.

In the psychiatric examination, reduction in self-care, tremor and muscle rigidity were noted. He was confused and his orientation was impaired. His vital signs were stable during follow-up in the emergency room, but it was learned that an ampoule of metamizole had been injected because of high fever (39.5 °C). Creatine kinase level was 1895 U/L [reference range: 0–199]. The white blood cell count was 13,750/mm^3^ [reference range: 4000–10,000]; the aspartate aminotransferase and alanine aminotransferase values were 55 IU/L [reference range: 0–32] and 45 IU/L [reference range: 0–33], respectively. His renal functions and serum electrolytes were within normal range. Electrocardiogram showed sinus tachycardia.

**Figure 1.**
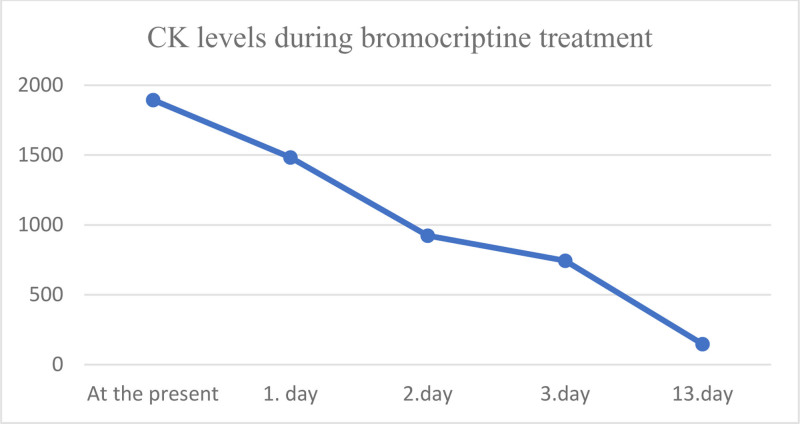
Creatinine kinase levels during bromocriptine treatment.

The patient was diagnosed as NMS and was referred to our psychiatry clinic. Bromocriptine 10 mg/day was initiated. Vital signs were stable and muscle rigidity and creatine kinase levels began to decrease (CK level 1483 U/L on the 1st day, 923 U/L on the 2nd day, 743 U/L on the 3rd day) on the second day of the treatment (Fig. 1). However, delirium and agitation continued. Lorazepam treatment (1 mg/day) was given for delirium for 1 day; however, as the patient’s symptoms worsened, lorazepam was stopped, and low dose amisulpride (200 mg/day) was started for delirium with agitation and administered for 13 days. Bromocriptine treatment was also continued for 13 days. With this treatment, the patient’s delirium improved and the patient became stable. CK level, aspartate aminotransferase level, alanine aminotransferase level and white blood cell decreased to normal values (Table 1). The patient was discharged from the hospital after 14 days of treatment with amisulpride 400 mg/day.

**Table 1:** Patient’s laboratory test results.

Variables	At presentation	At follow up	Normal range
White blood cell count (mm^3^)	13,750/mm^3^	5320/mm^3^	4000–10,000/mm^3^
Creatinine kinase (U/L)	1895 U/L	148 U/L	<199 U/L
Aspartate aminotransferase (IU/L)	55 IU/L	25 IU/L	<32 IU/L
Alanine aminotransferase (IU/L)	45 IU/L	27 IU/L	<33 IU/L

## 3. Discussion

Paliperidone is an atypical antipsychotic used in the treatment of schizophrenia. Oral and long-acting injectable (LAI) formulations (1-month and 3-month) are available.^[6]^ NMS associated with paliperidone has been reported very rarely in the literature. Duggal^[7]^ reported that he could not find any publications in PubMed search with the keywords “neuroleptic malignant syndrome” and “paliperidone” in June 2007. As of May 2023, a small number of publications as case reports in the search which were conducted with the same keywords is reached. Kane et al^[8]^ in their Janssen clinical trial database, detected a single case of NMS, when they examined 5008 patients using paliperidone.

The first known case of paliperidone-induced NMS was presented by Duggal.^[7]^ The case presented in this report (a 63-year-old, female patient) was admitted to the psychiatric unit for exacerbation of schizophrenia. The patient was started on oral paliperidone 6 mg/day and the drug was increased to 9 mg/day on the 5th day. She started to complain of increased stiffness, 6 days after starting oral paliperidone. Cogwheel rigidity, dysarthria, confusion, diaphoresis, tachycardia, labile blood pressure, a mild increase in temperature, a modest elevation in creatine phosphokinase (313 U/L) were observed in the patient in the following days, and paliperidone was discontinued with the diagnosis of NMS and supportive treatment was started. Mantas et al^[9]^ reported the second case of paliperidone-induced NMS in the literature. A 24-year-old male patient developed symptoms of NMS sometime after the paliperidone dose was increased from 6 mg/day to 9 mg/day. It was stated that the patient presented in this report did not meet all the criteria for NMS, therefore it was noted that the presentation may be variable and atypical during the use of atypical antipsychotics. Han et al^[10]^ reported NMS in a 58-year-old female patient with schizophrenia. In that case report, unlike the 2 cases mentioned above, although paliperidone was gradually increased by starting at a lower dose (from 3 mg/day to 6 mg/day), NMS developed and her CK level was quite high (26,791 U/L). Teng and Lane^[11]^ reported that NMS developed in a 32-year-old male patient who was switched from risperidone (4 mg/day) to low-dose paliperidone (3 mg/day), and noted that there was no certainty about dose adjustment when switching between antipsychotics. Nayak et al^[12]^ reported NMS following the use of oral paliperidone (3 mg b.i.d.) in a 13-year-old boy and noted that this undesirable side effect can also be seen in pediatric patients. Özdemir et al^[13]^ presented a 22-year-old male patient who developed NMS after the addition of oral paliperidone (6 mg/day) to amisulpride and withdrawal of clozapine, and emphasized the importance of early diagnosis. Langley-DeGroot et al^[14]^ presented a case (61 years old male patient) associated with LAI paliperidone and reported that bromocriptine is beneficial in controlling hyperthermia and that pharmacological treatment and early intervention may be helpful in stopping the progression of the syndrome. Kaur et al^[15]^ pointed out in their case report that side effects can be seen together during the use of antipsychotics. They reported syndrome of inappropriate antidiuretic hormone and rhabdomyolysis in addition to NMS in relation to the use of paliperidone in a 35-year-old male patient. Agarwal et al^[16]^ presented a 44-year-old male schizophrenic patient who had previously been given depot paliperidone and was diagnosed with NMS while on clozapine treatment. It was noted that this patient had an atypical presentation because no fever was observed throughout the process. Edinoff et al^[17]^ reported an NMS case developed as a result of atypical antipsychotic treatment in a 34-year-old male patient with a diagnosis of schizophrenia. Two days after starting aripiprazole and risperidone, the patient had confusion and elevated CK levels (7101 U/L). After the drugs were stopped and his clinical condition improved with intravenous hydration therapy, the patient was started on quetiapine, lithium, and paliperidone, and after the second dose of paliperidone, his mental status changed and CK levels increased (4272 U/L). Among the clinical findings of NMS, only a change in mental status was observed in this patient, which drew attention to the importance of laboratory examination (CK level) for not missing the cases.

The pathophysiology of NMS is not fully known. Regardless of dose and mode of administration, every antipsychotic drug has the potential to cause NMS. However, it has been suggested that various drug-related, personal or environmental risk factors may predispose the development of NMS. It has been reported that factors such as the use of antipsychotics at high doses, with rapid dose escalation or in depot formulations, intravenous administration, and polypharmacy increase the risk of NMS.^[18]^ Factors such as advanced age, comorbidity, stress, being in a hot environment for a long time, dehydration, and electrolyte imbalance have been suggested to provide a basis to the development of NMS. In addition, genetic factors, family history of NMS and previous NMS seem to be risk factors.^[18]^

NMS is more common in men than women (male:female ratio is 2:1). It can affect patients of all ages; however, it has been reported that the risk of NMS is higher in men under 40 years of age. This situation has been attributed to the higher use of antipsychotics in males in this age group, adherence to treatment, and pharmacodynamic differences between genders.^[1,19]^

The clinical presentation and course of NMS is heterogeneous. NMS has 4 main groups of symptoms; hyperthermia (from a mild pyrexia to a body temperature above 42 °C), muscle rigidity (generalized or local), altered mental status (mild confusion, anxiety, agitation, delirium, coma), and autonomic instability (diaphoresis, cardiac arrhythmias, labile blood pressure, hypersalivation, nausea, and vomiting).^[1,18]^ The main laboratory finding is an increase in CK levels, typically over 1000 U/L. However, if significant muscle rigidity does not develop, CK may be within normal limits, especially in the initial period of NMS.^[1,18]^ It has been reported that cases associated with atypical antipsychotics may show atypical presentation, and the diagnosis may be missed in the absence of typical findings.^[1]^ In this case, symptoms from these 4 groups were present and the CK level was found to be 1895 U/L.

Malignant hyperpyrexia, serotonin syndrome, malignant catatonia, clozapine-induced hyperpyrexia, and anticholinergic delirium should be considered in the differential diagnosis of NMS.^[18]^ Early diagnosis and treatment are important for prognosis. It has been reported that mortality rates, which were around 25% to 30% a few decades ago, have decreased over the years. The reason for this is thought to be an increase in the awareness of physicians and early treatment.^[20]^ However, mortality of around 10% is reported in NMS patients despite appropriate treatment.^[1]^ Advanced age, infections, respiratory failure, renal failure, and heart failure are risk factors for mortality.^[1,21]^

The patient with suspected NMS should be followed closely in a well-equipped intensive care unit. Antipsychotic drugs should be discontinued immediately.^[18]^ Symptomatic treatment and general supportive treatment should be applied (such as rehydration, cooling, maintaining electrolyte balance). Benzodiazepines can be used to relax muscles, to treat agitation and catatonia.^[19]^ Dantrolene is recommended as a muscle relaxant and bromocriptine as a dopamine-agonist. Amantadine is also used as an alternative due to its dopaminergic and anticholinergic effects.^[19]^ When it is needed to start treatment for psychosis again, it is recommended that an antipsychotic other than the one causing the episode be administered after a drug-free period of at least 2 weeks after the NMS symptoms have resolved.^[19]^

In NMS patients with delirium, treatment decision for patient’s agitation is difficult for clinicians. In this case antipsychotic drugs could not be used for delirium treatment because of NMS. Although it was reported that high-dose lorazepam and diazepam can be used for treatment of NMS with delirium,^[22]^ benzodiazepines and anticholinergic drugs were not used for agitation because of the increased risk of delirium symptoms in this case. Low dose lorazepam treatment was started for agitation, but as his symptoms worsened, the patient was started on low dose amisulpride for delirium.

## 4. Limitations

One potential limitation of this case study is the inadequate reporting of previous treatments, responses, compliance, and adverse drug reactions. This could have been due to the patient’s schizophrenia and the difficulty in accurately recalling and reporting this information. Additionally, we were only able to gather limited information about the patient’s medical history from a small number of family members due to poor social support. Furthermore, limited information was obtained from follow-up records due to the patient’s lack of regular medication use and irregular psychiatric outpatient clinic control. These could have also affected the reliability of the information gathered. It is important to note this limitation in the study and consider the potential impact on the patient’s outcome. Finally, the study is limited in its ability to provide information about the patient’s post-discharge status due to low compliance with treatment. This could have affected the long-term outcomes of the patient and should be taken into consideration when interpreting the results of the study.

It would be beneficial for future studies to be conducted with larger samples or to use alternative methods for gathering information about the patient’s medical history.

## 5. Conclusion

NMS is a rare condition that is difficult to diagnose and can be fatal. NMS is a neurological emergency in patients using antipsychotics. Although its incidence is not common, it is important as it can be fatal or leave permanent sequelae if not diagnosed early and treated appropriately. It has a prominent place among adverse events due to its unpredictability and potential side effects. This idiosyncratic reaction is less common with the use of second-generation antipsychotics, and there are few cases in the literature describing this reaction with the use of atypical antipsychotics. One such atypical antipsychotic is paliperidone, an active metabolite of risperidone. In cases of treatment-resistant schizophrenia with poor drug adherence, long-acting injectable antipsychotics, such as paliperidone palmitate, may be considered as an option on an individual basis. This LAI form of paliperidone is effective for both acute and maintenance treatment of schizophrenia. However, it is important for clinicians to be aware that NMS is a rare side effect of paliperidone and there have been limited reports of NMS caused by the use of LAI atypical antipsychotics. As a second-generation antipsychotic, paliperidone has the potential to cause NMS, making it crucial for clinicians to closely monitor patients for this rare but potentially life-threatening condition. If patients using LAI atypical antipsychotics experience changes in body temperature, level of consciousness, or confusion, NMS should be considered and early diagnosis and pharmacological intervention should be implemented to prevent its progression. In addition to clinical monitoring, it may be helpful to also monitor serum sodium and CPK levels in patients. LAI antipsychotics are not rapidly eliminated from the body after administration to the patient. The case study presented in this report demonstrates that NMS can be managed symptomatically while the patient is receiving LAI antipsychotics. Furthermore, it emphasizes the importance of early diagnosis and pharmacological intervention in preventing the progression of potentially life-threatening complications of antipsychotic use, such as NMS.

Further studies, including case reports, are needed to provide more information for clinicians to make treatment decisions for challenging cases.

## Author contributions

**Conceptualization:** Gonca Ayse Unal.

**Data curation:** Gonca Ayse Unal.

**Formal analysis:** Gonca Ayse Unal.

**Funding acquisition:** Gonca Ayse Unal.

**Investigation:** Gonca Ayse Unal.

**Methodology:** Gonca Ayse Unal.

**Project administration:** Gonca Ayse Unal.

**Resources:** Gonca Ayse Unal.

**Software:** Gonca Ayse Unal.

**Supervision:** Gonca Ayse Unal.

**Validation:** Gonca Ayse Unal.

**Visualization:** Gonca Ayse Unal.

**Writing – original draft:** Gonca Ayse Unal.

**Writing – review & editing:** Gonca Ayse Unal.

## References

[1] Oruch R Pryme IF Engelsen BA Lund A. Neuroleptic malignant syndrome: an easily overlooked neurologic emergency. Neuropsychiatr Dis Treat. 2017;13:161–75. 28144147 10.2147/NDT.S118438 PMC5248946

[2] Haddad PM Dursun SM. Neurological complications of psychiatric drugs: clinical features and management. Hum Psychopharmacol. 2008;23(suppl 1):15–26. 18098217 10.1002/hup.918

[3] Strawn JR Keck PE Jr Caroff SN. Neuroleptic malignant syndrome. Am J Psychiatry. 2007;164:870–6. 17541044 10.1176/ajp.2007.164.6.870

[4] Trollor JN Chen X Sachdev PS. Neuroleptic malignant syndrome associated with atypical antipsychotic drugs. CNS Drugs. 2009;23:477–92. 19480467 10.2165/00023210-200923060-00003

[5] Stahl SM. Essential Psychopharmacology. 2nd ed. New York: Cambridge University Press; 2004: 401–458.

[6] Edinoff AN Doppalapudi PK Orellana C. Paliperidone 3-month injection for treatment of schizophrenia: a narrative review. Front Psychiatry. 2021;12:699748. 34621193 10.3389/fpsyt.2021.699748 PMC8490677

[7] Duggal HS. Possible neuroleptic malignant syndrome associated with paliperidone. J Neuropsychiatry Clin Neurosci. 2007;19:477–8. 18070861 10.1176/jnp.2007.19.4.477

[8] Kane JM Correll CU Delva N Gopal S Savitz A Mathews M. Low incidence of neuroleptic malignant syndrome associated with paliperidone palmitate long-acting injectable: a database report and case study. J Clin Psychopharmacol. 2019;39:180–2. 30811377 10.1097/JCP.0000000000001019 PMC6392211

[9] Mantas C Kalabokis G Goulia P Tourlakopoulos A Hyphantis T Mavreas V. Possible neuroleptic malignant syndrome during paliperidone administration: a case report. J Clin Psychopharmacol. 2010;30:89–91. 20075663 10.1097/JCP.0b013e3181c9c7ef

[10] Han C Lee SJ Pae CU. Paliperidone-associated atypical neuroleptic malignant syndrome: a case report. Prog Neuropsychopharmacol Biol Psychiatry. 2011;35:650–1. 21163319 10.1016/j.pnpbp.2010.12.006

[11] Teng PR Lane HY. Emergence of neuroleptic malignant syndrome while switching between risperidone and paliperidone. J Neuropsychiatry Clin Neurosci. 2011;23:E16–7. 10.1176/jnp.23.4.jnpe16 22231327

[12] Nayak RB Bhogale GS Patil NM Chate SS Pandurangi AA Shetageri VN. Paliperidone-induced neuroleptic malignant syndrome. J Neuropsychiatry Clin Neurosci. 2011;23:E14–5. 10.1176/jnp.23.1.jnpe14 21304113

[13] Özdemir A Aksoy-Poyraz C Kiliç-Yener E. Possible paliperidone-induced neuroleptic malignant syndrome: a case report. J Neuropsychiatry Clin Neurosci. 2012;24:E22–3. 10.1176/appi.neuropsych.11070156 23037666

[14] Langley-DeGroot M Joshi Y Lehman D Rao S. Atypical neuroleptic malignant syndrome associated with paliperidone long-acting injection: a case report. J Clin Psychopharmacol. 2016;36:277–9. 27043124 10.1097/JCP.0000000000000507

[15] Kaur J Kumar D Alfishawy M Lopez R Sachmechi I. Paliperidone inducing concomitantly syndrome of inappropriate antidiuretic hormone, neuroleptic malignant syndrome, and rhabdomyolysis. Case Rep Crit Care. 2016;2016:2587963. 27721999 10.1155/2016/2587963 PMC5045994

[16] Agarwal P Omoruyi A Perai KG MacDaid K Burton A. Neuroleptic malignant syndrome (NMS) on clozapine with a potential atypical interaction with paliperidone. Case Rep Psychiatry. 2021;2021:5584104. 34007505 10.1155/2021/5584104 PMC8102093

[17] Edinoff AN Mohammad-Amin H Odisho AS. Silent neuroleptic malignant syndrome: a case report of atypical antipsychotic induced elevation of creatinine kinase and altered mental status. Health Psychol Res. 2022;10:37530. 35999974 10.52965/001c.37530 PMC9392844

[18] Pileggi DJ Cook AM. Neuroleptic malignant syndrome. Ann Pharmacother. 2016;50:973–81. 27423483 10.1177/1060028016657553

[19] Velamoor R. Neuroleptic malignant syndrome: a neuro-psychiatric emergency: recognition, prevention, and management. Asian J Psychiatr. 2017;29:106–9. 29061403 10.1016/j.ajp.2017.05.004

[20] Ware MR Feller DB Hall KL. Neuroleptic malignant syndrome: diagnosis and management. Prim Care Companion CNS Disord. 2018;20:17r–02185. 10.4088/PCC.17r02185 29325237

[21] Guinart D Misawa F Rubio JM. A systematic review and pooled, patient-level analysis of predictors of mortality in neuroleptic malignant syndrome. Acta Psychiatr Scand. 2021;144:329–41. 34358327 10.1111/acps.13359

[22] Tsai MC Huang TL. Severe neuroleptic malignant syndrome: successful treatment with high-dose lorazepam and diazepam: a case report. Chang Gung Med J. 2010;33:576–80. 20979709

